# The Philosophical Approach: An Interview with Ford Doolittle

**DOI:** 10.1371/journal.pgen.1005173

**Published:** 2015-05-07

**Authors:** Jane Gitschier

**Affiliations:** Departments of Medicine and Pediatrics and Institute for Human Genetics, University of California San Francisco, San Francisco, California, United States of America

**Fig 1:**
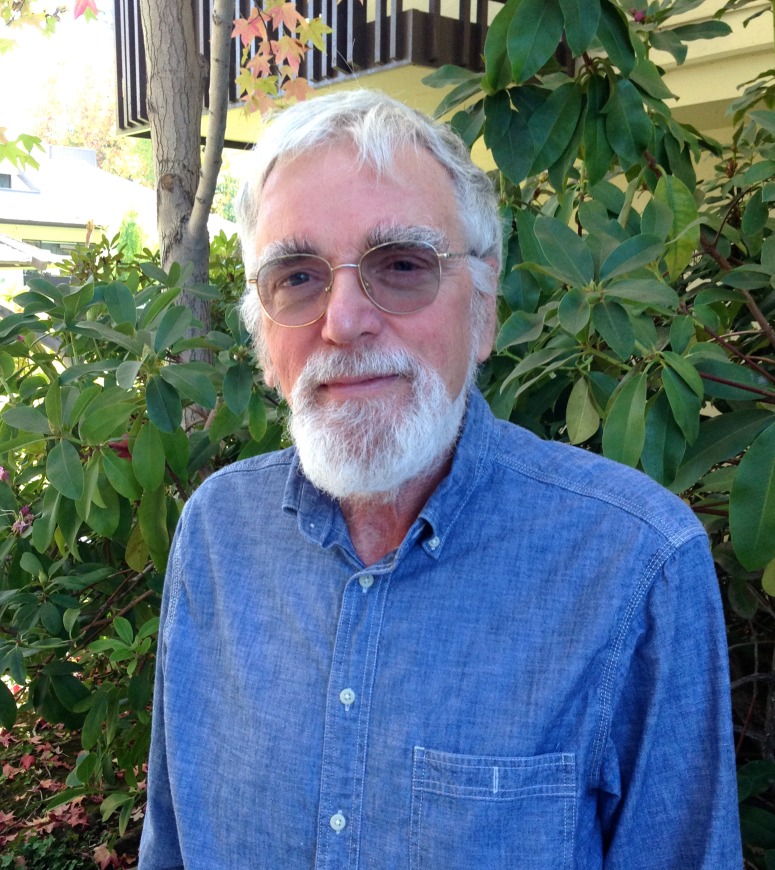
Ford Doolittle.

For years, the whiteboard in my office brimmed with ideas for potential interviewees. Names were erased when an interview was completed, and new names added when a particular topic piqued my interest. Some were arranged in a kind of Venn diagram by their fields, and one such cluster—concerning the origins of early life forms—included three deep thinkers: Lynn Margulis, whose 1967 paper articulated the endosymbiotic origin of mitochondria and chloroplasts; Carl Woese, whose attempts to classify prokaryotes based on ribosomal RNA cataloging led to his championing the new kingdom of Archaea in 1977; and Ford Doolittle (Fig 1), who provided evidence for Margulis’s hypothesis using Woese’s methods. Though Woese and Margulis are now deceased, Doolittle, I can attest, is very much alive and, dare I say, “kicking!” Over time, Doolittle has cogitated on a variety of intriguing evolutionary questions, including the origin of introns, the role of lateral gene transfer in speciation, and the meaning of “function.” He is always worth listening to.

Doolittle grew up in Champaign-Urbana, Illinois, the son of an art professor and now holding a BFA in photography himself. As a high-school friend of the late Sol Spiegelman’s son, he worked with Spiegelman in the summers. Following undergraduate studies at Harvard and graduate work with Charles Yanofsky at Stanford, he returned to Spiegelman’s lab for postdoctoral work and then joined Norman Pace when Spiegelman moved to New York.

Since then, Doolittle has spent his career in Halifax, Nova Scotia, at Dalhousie University. Last year he was awarded Canada’s highest scientific prize, the Gerhard Herzberg Gold Medal in Science and Engineering, and plans to pursue philosophical and biological questions with its largess. I was able to learn a lot more about these pursuits one warm morning in autumn when he was visiting the Bay Area.

**Gitschier:** Let’s start with Urbana and the atmosphere with Sol Spiegelman and Carl Woese.

**Doolittle:** Sol Spiegelman was a major figure who should have gotten the Nobel Prize, I think. For example, he was among the first to prove the existence and nature of messenger RNA. Woese was in Urbana when I did my first postdoc with Sol. Carl was a big influence on Sol’s students, because Sol was a remote figure in some ways—very inspirational, but very intimidating. But Carl was a drinking companion of Sol’s graduate students and postdocs. That’s how I got to know him and was primed to think about evolutionary problems.

**Gitschier:** And what were you all working on back then?

**Doolittle:** Sol was working on RNA tumor viruses and Q-beta RNA phage at that time. One of the things that got me interested in evolution was that Sol did these famous experiments on the Q-beta “mini-monsters.” You’re selecting for more rapidly replicating RNAs, so you get variants that are smaller and have better capacity for in vitro replication. It was kind of an in vitro evolution experiment, one of the earliest.

Ribosomal RNA precursors were *the* big thing in those days, partly because you could do polyacrylamide gels. You could pulse-label the RNA and slice the gel. In Norm Pace’s lab, I worked on—actually I was quite proud of this idea—testing whether 5S, 16S, and 23S RNA are transcriptionally linked. I thought that if I added label and rifampicin at the same time, we would get cessation of initiation of transcription but completion of transcription, so the specific activity would increase as you went along the transcript, and it did. We showed that 16, 23, and 5 were transcriptionally linked, in that order.

**Gitschier:** Then you started looking for jobs?

**Doolittle:** I applied for three, and I got offered one, which was at Dalhousie.

**Gitschier:** Why Canada?

**Doolittle:** My family had a summer place on Block Island [off Rhode Island]. My father grew up in New Haven. The Doolittles are from Connecticut, the first ones settling in 1640. I was looking for a place on the Atlantic. I wanted to be near the ocean, and I had these kind of romantic thoughts about moving to Nova Scotia because it is an idyllic sort of place. I have always been happy there, and it’s been a very good place for me.

**Gitschier:** Why is that?

**Doolittle:** I don’t actually like a lot of pressure, and Harvard and Stanford are great institutions, but they are not places where you can relax, particularly. At Dalhousie you don’t feel like you’re being threatened by your colleagues.

**Gitschier:** How long have you been there?

**Doolittle:** 43 years!

**Gitschier:** When you first moved to Canada in 1971, what were you working on?

**Doolittle:** I was going to continue with the biochemistry of ribosomal RNA maturation, but I didn’t really *want* to do that because I’m not a biochemist.

There was another fellow in the department working on blue-green algae, which are now called cyanobacteria. Back in those days, nobody even knew that they had ribosomes! These were primitive times! We probably assumed they had ribosomes, but nobody had done anything about ribosomal RNA precursors in cyanobacteria, so I thought this was a very low-hanging fruit. I started to look at their maturation patterns by pulse-labeling.

People were also vague about whether blue-greens were prokaryotes or not. The prokaryote/eukaryote distinction wasn’t very well drawn at that time. There were two theories. The earlier and still-dominant theory would be that bacteria evolved into blue-green algae, which were more complicated than bacteria, and blue-green algae evolved into eukaryotic algae, which are more complicated than prokaryotic algae, and everything else eukaryotic evolved from them. This was the “uralga” hypothesis.

Lynn Margulis was at Berkeley then. I didn’t read her ‘67 paper, but she published a book in 1970 called *The Origin of Eukaryotic Cells* which I did read. That was the re-invention, or the re-discovery, or the re-publication, of the endosymbiotic theory for the origin of chloroplasts and mitochondria, from cyanobacteria and bacteria, respectively.

**Gitschier:** So you were aware of her work around the time…

**Doolittle:** About the time that I started to work on cyanobacteria, but I didn’t actually have evolutionary ambitions until another fortuitous thing happened: Linda Bonen, who was a technician of Carl Woese, arrived in Halifax. She had helped Carl develop what was then called “oligonucleotide cataloging.” You take a culture and pump in something like 50 mCi of ^32^P, more ^32^P than my graduate students would have ever seen! You isolate the RNA and then you chop it up with ribonucleases. You do a two-dimensional fingerprint, and then you cut out those spots and do another two-dimensional fingerprint. Eventually you get what was called an oligonucleotide catalog: a list of all the oligonucleotides, from just a few bases up to maybe 15-bases long, all terminating in G because you use T1 ribonuclease as the first cut. It took three months to do a catalog from just one organism.

This was before reverse transcriptase sequencing from RNA, or cloning. And you didn’t get a complete sequence; all you had was this catalog. Then you can compare the catalog of one species to another species, and if they have lots of sequences in common, they are closely related species. Woese is the one who harnessed this for the purposes of phylogeny.

**Gitschier:** OK, back to Linda!

**Doolittle:** Linda Bonen showed up because her husband had also taken a job at Dalhousie, and Carl said, “Go look up Ford, he might have a job for you.” When she came along, I realized we could use this technique to test the endosymbiont hypothesis—a major evolutionary hypothesis.

The implication would then be that because chloroplasts have ribosomal RNA, that the ribosomal RNAs of chloroplasts and cyanobacteria would be much more closely related to each other than either would be to the ribosomal RNA of the nucleus.

**Gitschier:** What organism did you work on?

**Doolittle:** We studied an alga called *Porphyridium* because it was easy to grow and someone locally had cultures. And Carl did it pretty much at the same time, working on cyanobacteria with Ron Butow. But I think we did a better job of making the inference. Also, a colleague of mine, Michael Gray at Dalhousie, then acquired Linda Bonen as graduate student and they did the same thing with mitochondria. So I would say that Dalhousie deserves most of the credit for having proven the endosymbiont hypothesis, not that there aren’t multiple proofs.

**Gitschier:** Did you ever meet Lynn Margulis?

**Doolittle:** Oh, yeah, sure.

**Gitschier:** And, what was she like?

**Doolittle:** She was very bright, very vivacious, very outgoing. She had many extremely faithful students and supporters. But she was irascible, especially in her later years.

I was very fond of Lynn. She was very much an anti-establishment person. Evidence wasn’t all that important to her. Everybody gives her great credit for the endosymbiont hypothesis. She also always maintained that spirochetes were the ancestor of what she called the “undulipodia,” the eukaryotic flagellar apparatus, for which there is no evidence, nor ever was any evidence, yet she held onto that belief her whole life.

**Gitschier:** Let’s touch on Woese’s work, too. I remember that was a really big thing when I was in graduate school.

**Doolittle:** 1977 saw the publication of Archaea that got him on the first page of the *New York Times*. That was the same year as the discovery of introns. That was a big year.

**Gitschier:** Before I get to that year, can I ask you, Darwin…

**Doolittle:** Never worked with Darwin!

**Gitschier:** But Darwin, did he ever talk about microbes at all?

**Doolittle:** No.

**Gitschier:** I mean, it’s one thing to talk about the origin of species that we can all see and relate to. What was actually known about microbes at that time?

**Doolittle:** There’s a nice paper written by Maureen O’Malley, who was once a postdoc of mine, about what Darwin thought about microbes. People still believed in spontaneous generation in 1859. I don’t think he did, but whether or not bacteria were actually organisms at all or organisms in the usual sense of that word was still up for grabs in the 1880s.

People didn’t start trying to classify microbes until the latter part of the 19th century. And then, classification was pretty much an abyss until Woese came along.

That was one of Woese’s principal goals: to make a rational bacterial classification. Roger Stanier and Cornelius van Niel were the gurus at that time [mid-20th century]. I mean, Stanier [at Berkeley] was the Woese before Woese, and van Niel was Stanier’s supervisor at Hopkins. They had made a lot of attempts to classify bacteria, and basically there were two ways to do it: by the shape—we got the rods and we got cocci—or by the biochemistry—we got the ones that respire and the ones that photosynthesize, etc. If you try to map those two to each other, they don’t map, and people would argue about whether this or that trait was the more important.

In the early 1960s, Stanier, who wrote the principal textbook in microbiology in those days [*The Microbial World*] said, “Well, we can still identify them—this is *E*. *coli*, this is *Salmonella*, but we can’t figure out the relationships between the larger groups. And we’ll just give it up.”

But they thought that a bit prematurely because in the mid-1960s molecular phylogeny was being invented by Walter Fitch, Émile Zuckerkandl, and others. The notion that you could use protein sequences and make phylogenetic trees on the basis of sequence comparisons was coming along and a lot of people were doing that. But the problem with proteins is that it was taking people forever to sequence them in the old ways. Woese intended to put bacterial classification on rational grounds, based on ribosomal RNA sequence information.

**Gitschier:** And it took him, what, about ten years just going through…

**Doolittle:** A voice crying in the wilderness doing this! He did that and read most of them himself. It was a tedious enterprise. But it was fun, too. In some ways, it was like reading the Dead Sea Scrolls. It had a nice science fiction-y feel to it.

**Gitschier:** Since you brought up ‘77 and introns, I want to talk about that. You had a fantastic short paper in 1978 that proposed an “intron early” hypothesis [i.e., that introns were present in prokaryotes prior to the evolution of eukaryotes].

**Doolittle:** Here was the genesis of that paper: I was on sabbatical [at Harvard] in Wally Gilbert’s lab.

**Gitschier:** Why there?

**Doolittle:** Because I left Harvard with an enormous feeling of inferiority and stress.

**Gitschier:** Oh?

**Doolittle:** Well, it was hard for boys from the Middle West to feel really comfortable at Harvard. I mean, if you were a preppy, that was one thing, and if you went to the Bronx High School of Science, that was another, but the ones from the Middle West… I think I wanted to go back to Harvard on sabbatical to see whether it was really as aggressive, difficult, and stressful a place as I remember it being—which it is! The unique thing about Harvard is that it could be a nasty place and winds up making you feel that it is your fault! But it’s a superb place intellectually, and there is no question about the quality of the science done there.

So, here I was in Wally’s lab, and Wally comes back from visiting Susumu Tonegawa in Switzerland, and he’s all about introns. He gave a lab meeting and articulated his theory that introns served to facilitate exon shuffling, and that this is why eukaryotes had them. It allowed them to do this cool evolutionary stuff. He didn’t actually say where the introns came from.

At the same time, I was quite aware of what Woese was doing, so I knew that it was at least equally tenable that eukaryotes did not arise from within the prokaryotes but arose independently. The Woese view at that time would be that it was a tripartite world with eukaryotes, Bacteria, and Archaea all emerging from some kind of inchoate “ur” thing.

But what everybody but Woese actually believed was some version of the Margulis theory, which is that the prokaryotes arose first and then there was quite a lot of evolution of prokaryotes and then eukaryotes arose from within the prokaryotes.

So, if you believed that, and if you believed, as everybody believed then, that there are never going to be any introns in prokaryotes, then you would have to believe that introns were introduced into eukaryotes after they evolved from prokaryotes. That would be the only parsimonious explanation. That would have to have been what Wally would have believed, if he had thought about it in a phylogenetic context.

So I went home and I thought, well, it doesn’t make sense that eukaryotes would take on introns for the rather remote possibility of being able to evolve better in the future. Evolution doesn’t take things on because in the long term it will be glad it did. It must have some immediate advantage. But the alternative would be that introns were present in what Woese called the “progenote”—in the ancestral state—and had been retained in eukaryotes and lost in prokaryotes, because of streamlining.

So I wrote that paper overnight, and showed it to Wally and he said, “Oh, OK.” Wally wasn’t hostile, it just wasn’t what he had thought, but many were more skeptical.

Then after my paper appears, I get in the mail a long manuscript from Jim Darnell at Rockefeller, in which he argued pretty much the same thing, with more detail. And this was his paper that appeared in *Science*. Certainly Darnell’s parallel thinking was a big factor in getting this concept on the table. And then Wally basically embraced the idea. I don’t ever feel that Wally tried to steal my idea, but some people credit it to him, which is an oversimplification of what happened.

**Gitschier:** I see you’ve back-pedaled on this idea in recent years, but I don’t understand why. We now know that prokaryotes do have introns.

**Doolittle:** No spliceosomal introns. They do have group II and group I, and group IIs are almost certainly the ancestors of spliceosomal introns.

**Gitschier:** OK, well then let’s just talk about group II. What I’m trying to do is to defend your hypothesis!

**Doolittle:** Well, thanks, but what with the latest phylogenetic results, which show eukaryotes arising from within the Archaea, my “introns early” theory looks pretty unparsimonious. That theory, which Wally called “the exon theory of genes,” was that introns were left over from the joining together of short coding regions (exons) that arose first in some RNA world, before there were either prokaryotes or eukaryotes, indeed, before there were cells. There is no reason to believe that, and every reason to believe that group II introns, which are transposable elements, gave rise to spliceosomal introns at a much later stage, having been introduced into eukaryotes by the mitochondrial endosymbiont. They presumably spread as “selfish DNAs” throughout the eukaryotic nuclear genome, which did not have them before. I call this “introns late.” It occurred long after the time of the RNA world.

**Gitschier:** So how do you think spliceosomal introns arose?

**Doolittle:** OK, group II intron comes into a bar… and self-splices—but there are some variants that are dependent upon a maturase, a protein that binds to it, so I would say it was fortuitously bound in the beginning, but by its binding acts as a pre-suppressor for mutations in the RNA which would have otherwise incapacitated the RNA for self-splicing. Similarly, the intron becomes fragmented into the several snRNAs now involved in splicing, as Phil Sharp proposed in his aptly titled “Five Easy Pieces.” And once you have several of these pre-suppressive interactions, you can’t go back again, and the entire spliceosome, with its hundreds of proteins and its interacting RNAs, is the result of this kind of degenerative process of pre-suppression, not necessarily ever positively selected for.

I think most people would believe—“Oh, look at the spliceosome; it’s such a complicated structure. Isn’t nature wonderful?” And I would say, “Isn’t nature stupid?” It can’t avoid getting this kind of complexity because of these kinds of ratchet traps that it falls into.

That’s what we call “cellular bureaucracy.” And most people get that bureaucracy analogy because if you consider your university and how complicated its organization is… and you think, well if we could just start from scratch we could make a much better job of it! But we can’t start from scratch.

**Gitschier:** Let’s now talk about the “web of life.” How did you get onto that…

**Doolittle:** Hobby horse? I think it started in my lab with a postdoc named Jim Brown, who published a review with me in *Microbial and Molecular Biological Reviews* in 1997. He looked at the protein gene sequences for which bacterial, archaeal, and eukaryotic nuclear versions were all known, of which there weren’t so many. He asked how many trees supported what most people believed then, which was that Archaea and eukaryotes should be sister groups, and Bacteria should be a deeper branch.

**Gitschier:** Because of Woese?

**Doolittle:** Basically. But the fact is that different genes have different trees. The ones that Jim looked at were more or less equally divided between ones that showed Bacteria and Archaea being sisters, Bacteria and eukaryotes being sisters, and Archaea and eukaryotes being sisters.

That indicated to us that maybe lateral gene transfer was a more significant force than most people were willing to think. Some people *were* willing to think that, though. Peter Gogarten at the University of Connecticut would have been one of my heroes in this regard; he was early on pushing for there being more lateral gene transfer than people were willing to admit.

There certainly was a period during which if you did a phylogenetic tree and it didn’t work very well and you wanted to publish it, you’d say maybe lateral gene transfer was involved. It was the last resort of the impoverished imagination.

I think I got some notoriety with my 1999 *Science* review. It’s the one that has these figures that I hand-drew, which people often use still. It’s called “Phylogenetic classification and the universal tree.”

In 1999, we didn’t know very much yet. There were many individual cases where people were inferring lateral gene transfer and some irritating inability to disprove lateral gene transfer in other cases. I said, if it turns out to be as big as I think it’s going to turn out to be, then we just have to wonder whether a tree is the appropriate model. And I think that is expressed much more clearly in the 2007 *PNAS* paper with Eric Bapteste.

**Gitschier:** But now you’re off on another thing.

**Doolittle:** I’m off on function now.

**Gitschier:** Don’t abandon these things!

**Doolittle:** I come back to them.

I’m drifting off into philosophy in my old age. People have gotten quite heated over whether or not there is a tree of life. And really the question is what do you mean by the “tree of life?” And clearly—this is sort of Eugene Koonin’s position, which he calls the “forest of life”—there is a consensus signal.

If you make trees of all genes, it’s not completely random. Patterns emerge. And then you think, “Well duh, what the hell else do you expect?” Nobody would expect that lateral gene transfer wouldn’t respect geography or biochemistry or ecology or whatever.

There is no fact of the matter there: it’s just a question of what you want to believe. There is a consensus tree, there is a majority tree, there is some kind of central tendency of the data, but whether that central tendency reflects historical branching or instead reflects ecology is another question.

Imagine you started out with three separate populations of things, and they just started exchanging genes with each other, and population A changed genes with B more frequently than they do with C. Eventually, then, most of the genes that you might make a tree of would show A and B as being sister with each other and imply that there was a common A-B ancestor, but there need never have been a common A-B ancestor. It is possible that you could get quite a robust tree of life, but it doesn’t necessarily mean that the nodes in that tree correspond to ancestors in the history of life.

**Gitschier:** Now with all the many genome sequences available, what does Doolittle posit about the relationship between Bacteria, Archaea, and eukaryotes?

**Doolittle:** I think there are two groups of prokaryotes: Bacteria and Archaea. They are not well defined, and there are many genes that are derived from lateral gene transfer from bacteria into archaea, somewhat fewer in the other direction. So to really say that “this bug is an archaeon” when the majority of its genes are actually bacterial, what you really mean is that you are privileging the ribosomal RNA as the definer. And that is what people do, so I will let them do that.

And then what people would believe, and I guess what I would believe, is that the eukaryotic transcriptional/translational machinery—the informational machinery in the eukaryotic cell—arose within the Archaea, more recently than the Bacteria and the Archaea diverged from each other. That would be the standard view.

But we think that a tremendous number of genes have been exchanged back and forth between bacteria and between bacteria and archaea, and also between bacteria and eukaryotes after eukaryotes arose from within the Archaea—so much transfer that it is really rather arbitrary to define these lineages by virtue of their transcriptional and translational machinery any more. Had Woese started looking at glycolysis enzymes, rather than ribosomal RNA, we might not even be talking this way.

There is a nice paper coming out in *Nature* by Bill Martin [Heidelberg] and others claiming that the various different archael groups arose by the importation of different bacterial genes at different times in the past; so in a sense, the Archaea are the universal acceptors.

One of the poster children of the Archaea has been the halobacteria; they live in the salt and they are pink. They are aerobes—they have to be because they are sitting there on the top of water—and we’ve known for a very long time that the cytochromes and other aerobic things are bacterial in nature in those archaea. Martin and his colleagues show that they have accepted a bunch of bacterial genes for aerobic capacity. The nice thing about that view is that it makes eukaryotes just another group of Archaea that accepted a bunch of bacterial genes.

**Gitschier:** I want to close with what you describe as your “latest rant.” How did you get on function?

**Doolittle:** Well, I’ve always been on that.

Back in 1980, people were talking about transposable elements as if their function was to speed evolution; that they exist because of their future utility. And I’ve never liked that kind of idea. I didn’t like it in terms of introns. And Dawkins had just published *The Selfish Gene* in 1978.

Carmen Sapienza, a student of mine who now works on eukaryotic imprinting, and I wrote a paper which was rejected by *Science* after seven referees. But we heard that Leslie Orgel and Francis Crick were working on something like this, so we sent it to them. They said, “If you submit it to *Nature*, we will tell *Nature* not to publish ours without publishing yours, and to publish yours first,” etc., which was very nice.

That paper, seemingly now very simplistic, said you don’t need to suppose that transposable elements are there for the purpose of speeding evolution. These are selfish things, and natural selection will favor such elements that can make copies of themselves in genomes and then spread horizontally to other genomes within the species. These are basically parasites. I think many people would now accept this, but it was radical at the time.

People don’t like to think that the human genome has junk in it. This came back when the ENCODE papers came out a few years ago and were touted as spelling the “demise of junk DNA.” That got my dander up.

I wrote a perspective in *PNAS*, and Dan Graur had a much more vituperative thing in *Genome Biology and Evolution*. I don’t think the ENCODE people have given up; they had a kind of semi-apology in *PNAS*, which wasn’t really an apology.

It is the same as the tree of life issue, but until we actually have some agreement about what we mean by *words* we are going to get into these arguments, and in my mind, there are two devastating things you can say about the ENCODE people.

One is that they completely ignored all that history about junk DNA and selfish DNA. There was a huge body of evidence that excess DNA might serve some structural role in the chromosomes, but not informational. They also ignored what philosophers of biology have spent a lot of time asking: what do you mean by “function?” And you can mean one of two things: we might mean either what natural selection favored, which is what I think most biologists mean, or we might mean what it does. Some people might say, “Well the function of this gene is in the development of cancer,” but they don’t really mean that natural selection put it there so that it would cause cancer. These are not-so-subtle differences.

I think many molecular biologists and genomicists, in particular, think that each and every nucleotide is there for a reason, that we are perfect organisms. It is almost as if we were still theists thinking God doesn’t make junk; we just now think natural selection doesn’t make junk. I think there is a deep issue about the extent to which we are noisy creatures and the extent to which we are finely honed machines. I think the latter view informs much of genomics, and I think it is false.

ENCODE wouldn’t have got funded had they said 80% of the human genome is just junk, transposable elements.

**Gitschier:** It is justifying itself, post hoc. They are the big players with a lot of money. It’s like a machine—“We *can* do it, so let’s just do it!”

**Doolittle:** It’s a juggernaut is what you are saying.

My other objection is that it is false ontology. I think all of our science suffers not only from the big science motivation, but from what I call “positivism.”

A couple of times we submitted papers saying, “Everybody’s doing something this way, and it doesn’t work, and it is wrong to do it this way.” And *Nature* would write back, “We’re not interested in negative reports like this. What does work?” And we say, “We don’t give a damn what *does* work, it is important to know that what people are doing now is not working.”

There is no critique in science, very little. You can’t actually say, “This doesn’t mean what people say it means.” You’ve got to be “positive;” you’ve got to be moving the program forward all the time. I don’t think that is right.

Now, and down the road, we’re going to tackle directly relevant questions, like what is the meaning of function in the concept of genomics? There are legitimate evolutionary constructs in which you can address transposable elements, and people have not really explored that. Questions about the tree of life, again, and some of the questions we’ve been through are things that continue to interest me and which have a strong philosophical component as well as a data-related component. That’s what I’m interested in pursuing.

